# Effects of CK2β subunit down-regulation on Akt signalling in HK-2 renal cells

**DOI:** 10.1371/journal.pone.0227340

**Published:** 2020-01-07

**Authors:** Estefania Alcaraz, Jordi Vilardell, Christian Borgo, Eduard Sarró, Maria Plana, Oriano Marin, Lorenzo A. Pinna, José R. Bayascas, Anna Meseguer, Mauro Salvi, Emilio Itarte, Maria Ruzzene

**Affiliations:** 1 Departament de Bioquímica i Biologia Molecular, Unitat de Bioquímica Facultat de Biociències, Universitat Autònoma de Barcelona, Bellaterra (Barcelona) Spain; 2 Department of Biomedical Sciences, University of Padova, Padova, Italy; 3 Fisiopatología Renal, CIBBIM-Nanomedicine, VHIR, Barcelona, Spain; 4 Centro de Investigación Biomédica en Red en Bioingeniería, Biomateriales y Nanomedicina (CIBER-BBN), Cerdanyola del Vallès, Barcelona, Spain; 5 CNR Neuroscience Institute, Padova, Italy; 6 Departament de Bioquimica i Biologia Molecular, Unitat de Bioquímica de Medicina, Universitat Autònoma de Barcelona, Bellaterra (Barcelona) Spain; 7 Institut de Neurociències, Universitat Autònoma de Barcelona, Barcelona, Spain; 8 Red de Investigación Renal (REDINREN), Instituto de Salud Carlos III-FEDER, Madrid, Spain; University of Pittsburgh School of Medicine, UNITED STATES

## Abstract

The PI3K/Akt pathway is interconnected to protein kinase CK2, which directly phosphorylates Akt1 at S129. We have previously found that, in HK-2 renal cells, downregulation of the CK2 regulatory subunit β (shCK2β cells) reduces S129 Akt phosphorylation. Here, we investigated in more details how the different CK2 isoforms impact on Akt and other signaling pathways.

We found that all CK2 isoforms phosphorylate S129 *in vitro*, independently of CK2β. However, in HK-2 cells the dependence on CK2β was confirmed by rescue experiments (CK2β re-expression in shCK2β HK-2 cells), suggesting the presence of additional components that drive Akt recognition by CK2 in cells. We also found that CK2β downregulation altered the phosphorylation ratio between the two canonical Akt activation sites (pT308 strongly reduced, pS473 slightly increased) in HK-2 cells. Similar results were found in other cell lines where CK2β was stably knocked out by CRISPR-Cas9 technology. The phosphorylation of rpS6 S235/S236, a downstream effector of Akt, was strongly reduced in shCK2β HK-2 cells, while the phosphorylation of two Akt direct targets, PRAS40 T246 and GSK3β S9, was increased. Differently to what observed in response to CK2β down-regulation, the chemical inhibition of CK2 activity by cell treatment with the specific inhibitor CX-4945 reduced both the Akt canonical sites, pT308 and pS473. In CX-4945-treated cells, the changes in rpS6 pS235/S236 and GSK3β pS9 mirrored those induced by CK2β knock-down (reduction and slight increase, respectively); on the contrary, the effect on PRAS40 pT246 phosphorylation was sharply different, being strongly reduced by CK2 inhibition; this suggests that this Akt target might be dependent on Akt pS473 status in HK-2 cells.

Since PI3K/Akt and ERK1/2/p90rsk pathways are known to be interconnected and both modulated by CK2, with GSK3β pS9 representing a convergent point, we investigated if ERK1/2/p90rsk signaling was affected by CK2β knock-down and CX-4945 treatment in HK-2 cells. We found that p90rsk was insensitive to any kind of CK2 targeting; therefore, the observation that, similarly, GSK3β pS9 was not reduced by CK2 blockade suggests that GSK3β phosphorylation is mainly under the control of p90rsk in these cells. However, we found that the PI3K inhibitor LY294002 reduced GSK3β pS9, and concomitantly decreased Snail1 levels (a GSK3β target and Epithelial-to-Mesenchymal transition marker). The effects of LY294002 were observed also in CK2β-downregulated cells, suggesting that reducing GSK3β pS9 could be a strategy to control Snail1 levels in any situation where CK2β is defective, as possibly occurring in cancer cells.

## 1. Introduction

Protein kinase CK2 is a constitutively active serine/threonine kinase present in eukaryotic cells which acts on a large number of protein substrates involved in a plethora of cellular functions [1]. In mammals, CK2 is mainly present as a tetramer, composed of two catalytic (CK2α and CK2α’) and a dimer of two regulatory subunits (CK2β), giving rise to different forms of the holoenzyme (α_2_β_2_, αα’β_2_ and α’_2_β_2_) [2–4]. However, unbalanced expression of CK2α/CK2α’ and CK2β have been detected in different mammalian tissues and human cancer cells, pointing to the existence of free catalytic or regulatory subunits [1,5–7]. CK2α and CK2α’ show high similarity in their structural and *in vitro* enzymatic characteristics, but genetic and other functional experiments suggest that they may have marked different *in vivo* functions [8]. On the other hand, CK2β might influence substrate specificity, since there are protein substrates whose phosphorylation is specifically catalysed by either the free catalytic subunits or CK2 holoenzyme [3]. Moreover, CK2β also binds to a wide range of other cellular proteins and acts as a regulatory binding partner of certain protein kinases [9–11], suggesting that it can exert cellular functions other than its incorporation into CK2 holoenzyme.

The involvement of CK2 as an integral component of a signalling pathway has not been proven, yet. Rather, CK2 acts laterally by modulating the activity of a variety of signalling proteins and contributes to maintain cellular signalling homeostasis [12]. Of special relevance for this work, the RAF-MEK-ERK and PI3K/Akt pathways have been reported. On RAF-MEK-ERK, it has been shown that CK2α overexpression cells resulted in MEK deactivation in NIH 3T3 [13], and CK2α and CK2α' silencing led to increases in ERK1/2 phosphorylation in human glioblastoma cell lines [14]. Concerning the PI3K/Akt pathway, a very intricate network of connections to CK2 is known [15]. CK2 directly phosphorylates Akt1 at S129; this promotes Akt activation, also favouring a phosphorylated state of the Akt T308 activating site [16,17]. However, this only applies to Akt1 isoform, since we found that the other main isoform Akt2 is not target by CK2 [18,19] (while little is known on Akt3, which is more tissue-specifically expressed, and does not contain a site homolog to Akt1 S129). There are many other levels of CK2 intervention on PI3K/Akt pathway. CK2 phosphorylates PTEN, the lipid phosphatase which reverses the PI3K signalling by dephosphorylating PIP_3_ to PIP_2_; CK2-dependent phosphorylation of PTEN has a double and counterintuitive effects, because it increases PTEN stability but also inhibits its lipid phosphatase activity [20,21]. Other connecting points between CK2 and Akt signalling are represented by some mTORC1 and mTORC2 components phosphorylated by CK2 [15]. mTORC2 is the responsible of Akt S473 phosphorylation. This site, along with T308, have to be phosphorylated to produce Akt maximal activation [22]. T308 is targeted exclusively by PDK1, while S473, beside mTORC2, can be phosphorylated by other protein kinases [22,23]. Further complexity is generated by the cross-talk between the two activation sites, being pS473 potentially able to affect the PDK1-dependent T308 phosphorylation [24]. The ratio of pT308/pS473 is crucial, because it possibly impacts on Akt target specification. Several studies reported on this ratio, with different observations, possibly due to cell type-dependent events and the downregulation loop existing between mTORC1/S6K1 and mTORC2 [24–27].

Once activated, Akt phosphorylates the tuberous sclerosis complex 2 (TSC2) within the TSC1/TSC2/TBC1D7 complex and the Proline-Rich Akt Substrate of 40 kDa (PRAS40), leading to the activation of mTORC1, which conveys the signal downstream to the p70 ribosomal S6 protein kinase (S6K1) and other cellular targets [22]. Activation of S6K1 leads to phosphorylation of ribosomal S6 protein (rpS6) which is used as distal reporter of the activation of this signalling cascade.

Glycogen Synthase Kinase 3β (GSK3β) is another substrate of Akt, which phosphorylates it at Ser9 and causes GSK3β inactivation. GSK3β regulates a large number of downstream targets involved in a wide range of cellular processes [28], and represents an alternative branch to mTORC1 to convey the signal downstream of Akt. However, GSK3β activity is not only controlled by the PI3K/Akt signalling pathway: p90 ribosomal S6 protein kinase (p90rsk, also known as RSK) can also phosphorylate GSK3β at the inactivating Ser9 [29], making it difficult to discern the actual contribution of Akt in the control of the many GSK3β targets [22]. p90rsk is activated through its sequential phosphorylation by ERK1/2 and PDK1, and can also phosphorylate rpS6 and TSC2, adding further complexity to the cross-talk between the ERK1/2 and the PI3K/Akt pathways [22,30].

Protein kinase CK2 is known to cooperate with GSK3β in the phosphorylation of Snail1, a transcription factor that plays a key role to initialize and maintain the epithelial-to-mesenchymal transition (EMT) process [31]. Hierarchical phosphorylation operated by CK2 holoenzyme and GSK3β targets Snail1 to degradation; downregulation of CK2β is sufficient to stabilize Snail1 and to induce an EMT-like phenotype in normal human breast epithelial MCF10A cells [32]. In a previous work, we observed that the stable downregulation of CK2β increased Snail1 protein levels, indicative of EMT, in renal proximal tubular (HK-2) cells [7]. We also found that downregulation of CK2β decreased Akt1 Ser129 phosphorylation in HK-2. In the present work we have further studied the impact of CK2β on Akt and ERK1/2 pathways in the HK-2, also in comparison to chemical targeting of CK2.

## 2. Materials and methods

### 2.1 Cell lines, transfections, knock out and treatments

Proximal tubule epithelial cell line HK2 (CRL-2190^TM^), were obtained from the American Type Culture Collection (ATCC). HK-2, HEK293T (human embryo kidney fibroblasts), C2C12 (murine myogenic cells) and GN11 (murine GnRH neuronal cells) cells were cultured in Dulbecco’s Modified Eagle’s Medium (DMEM) supplemented with 10% fetal bovine serum (FBS), 1% (v/v) L-Glutamine, 1 mM sodium pyruvate, 1% (v/v) Streptomycin and Penicillin at 37°C in an incubator. Stably-silenced cell lines for either CK2α or CK2β subunits (shCK2α and shCK2β, respectively) as well as control (shCV) cell lines were generated as indicated before [7]. HK-2 stably-silenced cell lines were maintained in complete DMEM medium supplemented with 1 μg/mL of puromycin in a cell incubator. Transient transfection of HK-2 cells with the human codon-optimized CK2β pCMV-HA vector was carried out as reported previously [7] using Metafectene PRO (Biontex Laboratories, Germany).

For the CK2β siRNA silencing experiments, HK-2 and HEK293T cells were transfected with 30 nM CK2β specific siRNA (sequence target: 5'-GCCATGGTGAAGCTCTACT-3') or scrambled siRNA using the GenMute siRNA Transfection Reagent (SignaGen Laboratories, Rockville, MD, USA) according to the manufacturer’s recommendations.

Cells were lysed 48 h after transfection by scraping in the lysis buffer and analysed by immunoblotting.

CRISPR-Cas9 CK2β knock out (KO) in C2C12 and GN11 cells was performed as described in [33]. Briefly, C2C12 and GN11 cells were transfected with all-in-one pD1301-AD plasmid expressing Cas9-DasherGFP and the sgRNA guide to target CK2β subunit and Lipofectamine 3000 (ThermoFisher Scientific, Waltham, MA, USA) according to manufacturing instructions. Cells were single-cell sorted in 96-well plates using fluorescence-activated cell sorting (FACS) with a FACSAria II cell sorter (BD Biosciences) 48 h after transfection and expanded to obtain individual clones. The western blotting analysis were used to verify the absence of CK2β.

Cell treatment with CX-4945 (Selleckchem) was performed in DMEM medium supplemented with 10% FBS as indicated in the figure legends.

### 2.2 Cell lysates and western blot analysis

Cell lines extracts were obtained from cells at 70–80% confluence. Cells were washed with PBS and lysed using the cell lysis buffer (50 mM Tris/HCl pH 7.4, 150 mM NaCl, 1% triton-X-100, 1 mM DTT, 1 mM PMSF, 1 mM EDTA, 25 mM NaF, 0,2 mM Na_2_VO_3_, 2 mM PPi, 1 μg/mL protease inhibitors (leupeptin, benzamidin, aprotinin, pepstatin)). Protein concentrations of the extracts were determined by the colorimetric method of Bradford according to the manufacturer’s instructions (BioRad). Equal amounts of proteins were loaded in 10% SDS polyacrylamide gels (SDS-PAGE), subjected to electrophoresis and subsequently electrotransfered to polyvinylidene fluoride membranes (PVDF, Immobilion P, Millipore). Unspecific proteins were blocked in 5% non-fat dry milk in TTBS (50 mM Tris/HCl, pH 7.4, 150 mM NaCl and 0.1% Tween-20) for 1h at room temperature. Membranes were then incubated overnight with the adequate primary antibodies at 4°C, rinsed in TTBS and incubated with their respective secondary antibodies conjugated to horseradish peroxidase (HRP). The signal was generated using Lumi-light Western blotting substrate (Roche, GE) and detected by Amersham Hyperfilm ECL (GE-Healthcare) or Chemidoc MP Image System (Bio-Rad, Hercules, CA). Primary antibodies used were: anti-CK2α 1AD9 (05–1431, Millipore; it detects also α’ [34], anti-CK2β 6D5 (sc-12739, Santa Cruz Biotechnology), anti-p-Akt1(S129) (ab133458, EPR6150, Abcam), anti-p-Akt(308) (9275, Cell Signaling), anti-p-Akt(S473) (9271, Cell Signaling), anti-Akt1/2/3 (9272, Cell Signaling), anti-Akt1 (2938, C73H10, Cell Signaling), anti-Akt2 (3063, D6G4, Cell Signaling), anti-p-GSK3β(S9) (9336, Cell Signaling), anti-GSK3β (610201, 7/GSK-3b, BD Transduction Laboratories), anti-p-PRAS40(2997,C77D7, Cell Signaling), anti-PRAS40 (raised in sheep against the peptide DLPRPRLNTSDFQKLKRKY corresponding to residues 238 to 256 of human PRAS40), anti-p-rpS6 (Ser235/236, D57.2.2E, Cell Signaling), anti-rpS6 (2217, 5G10, Cell Signaling), anti-p-ERK1/2(T202/Y204) (9101, Cell Signaling), anti-p-PDK1(S241) (3061, Cell Signaling), anti-ERK1/2 (610124, Transduction Lab), anti-p-p90rsk (12445 Santa Cruz Biotechnology), anti-p90rsk (9355, Cell Signaling), anti-Snail1 (3895, L70G2, Cell Signaling), anti-phospho-CK2 substrates (8783, P-S/T3-100, Cell Signaling), anti-β-actin (sc-47778, C4, Santa Cruz Biotechnology) and anti-β-tubulin (2146, Cell Signaling). Anti-eIF2β antibodies were raised in rabbits and used as described previously [35]. Anti-p-eIF2β(S2) antibody was also raised in rabbits immunized with the peptide MpSGDEMIFDPC (p-eIF2β, residues 1–10) and the immunoglobulin fraction was obtained from sera by Protein A–agarose chromatography. Secondary antibodies were obtained from Bio-Rad: IgG Goat Anti-Rabbit IgG (H+L)-HRP conjugate (170–6515, BioRad), IgG Rabbit Anti-Sheep (H+L)-HRP conjugate (172–1017) and IgG Goat-Anti Mouse IgG (H+L)-HRP conjugate (170–6516, BioRad).

### 2.3 CK2 activity assays and Akt phosphorylation in vitro

Purified human recombinant CK2 forms (CK2α, CK2α’ or CK2α2β2) were expressed, purified and kindly donated by Dr. Stefania Sarno (Padova, Italy). The protein kinase activity of the recombinant CK2 forms was determined using the CK2 specific peptide (CK2-tide: RRRADDSDDDDD) as substrate, in the presence of phosphorylation mixture as described previously [36]. In experiments where the different CK2 isoforms were compared, we used the same units of each isoform (where 1 unit is the amount of enzyme needed to transfer 1 pmol of Pi to the peptide substrate CK2-tide in 1 minute). Purified recombinant Akt (0.2–0.5 μg) was incubated with 5–20 units of human recombinant CK2 (CK2α, CK2α’ or CK2 α_2_β_2_) in the presence of 50 mM Tris-HCl, pH 7.5, 12 mM MgCl_2_, 0.1 M NaCl, 10 μM [γ-^33^P]ATP (approx. 1500 cpm/pmol), in a final volume of 20 μL. After 10 min incubation at 30°C, reactions were stopped by the addition of 20 μL Laemmli buffer and heating at 100°C. The samples were run on 10% SDS polyacrylamide gels (SDS-PAGE), then the gel was stained with Comassie, dried and analysed for radioactivity by digital autoradiography (CyclonePlus Storage Phosphor System, PerkinElmer). Active Akt1 (phosphorylated at T308 and S473) and inactive Akt1 were purchased from Active Motif. The protein kinase activity of the recombinant CK2 forms was also analysed by the phosphorylation of β-casein under the same condition used for Akt1 phosphorylation.

### 2.4 Statistical analysis

The number of experiments (n) is indicated in each figure legend. Statistical Analysis was performed using GraphPad Prism 5 program for Windows. Mann-Whitney (when n>3) or Student’s t-test (when n≤3) were used to analyse Statistical significance, setting (*) p<0.05, (**) p <0.01, and (***) p<0.001.

## 3. Results

### 3.1 Free CK2α and CK2α’ phosphorylate Akt1 ‘in vitro’ but CK2β is also required for its phosphorylation in cultured HK-2 cells

**Fig 1:**
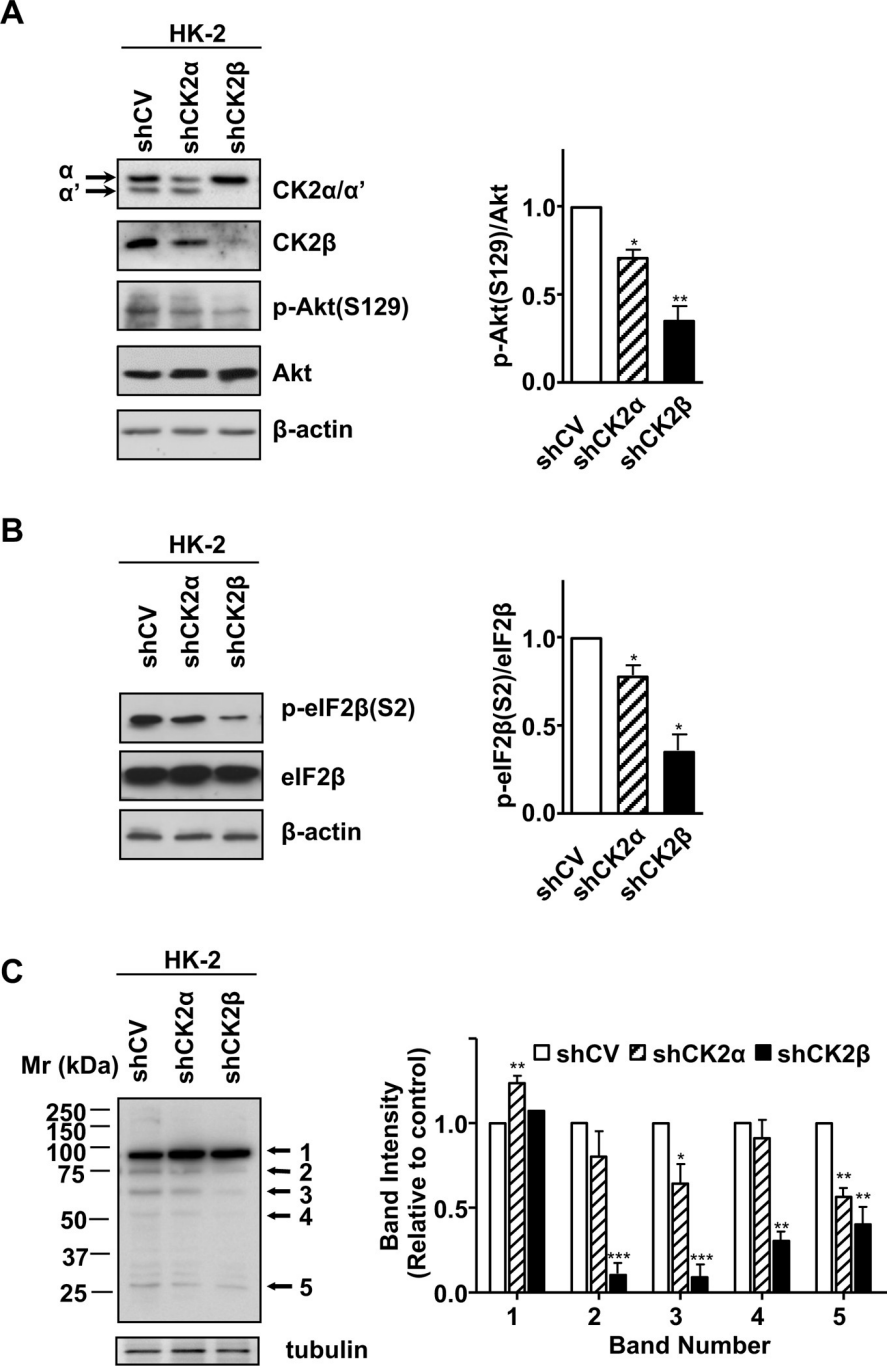
CK2 expression and activity in response to CK2α and CK2β downregulation in HK-2 cells. (A) HK-2 control (shCV) and silenced cells (shCK2α and shCK2β) were analysed for the expression of each CK2 subunit (α,α’ or β) and for the phosphorylation state of the CK2 target Akt S129. The 1AD9 anti-CK2α antibody detects also CK2α’; the arrows indicate the migration of each catalytic isoform. (B) Detection of total and phosphorylated eIF2β(S2) using specific antibodies. β-actin was used as a loading control. (C) The phosphorylation of endogenous CK2 substrates were analysed in lysates of HK-2 control (shCV) and silenced cells (shCK2α and shCK2β) by Western blot using Phospho-(Ser/Thr) CK2 Substrate (P-S/T3-100) antibody. Arrows indicate the bands that are more affected by CK2β down-regulation, whose quantification is shown in the bar graph. Tubulin was used as a loading control. A representative Western blot is shown in each case, while quantification (means ± SEM) is shown in the bar graphs. n = 4 for panel (A) and (B), n = 3 for panel (C).

HK-2 cell lines stably expressing shRNA for silencing either CK2α or CK2β were generated, as previously indicated [7]. The effects of silencing on the CK2 subunit amount was analyzed by Western blots of cell lysates, with antibodies recognizing CK2α/α’ and CK2β (Fig 1A). As already reported and similarly to other cell lines [7,37], the decrease in CK2α levels caused by stable expression of shCK2α was accompanied by a substantial decrease also of CK2β subunit. On the other hand, the stable expression of shCK2β was accompanied by a strong reduction of CK2β and CK2α’ but it did not decrease CK2α levels (Fig 1A). Therefore, a decreased amount of holoenzyme is expected in response to both silencing, but in particular in shCK2β cells.

Our previous study has shown that free CK2α is efficient in phosphorylating Akt1 in vitro [16]. However, we have also found that down-regulation of CK2β in cells (shCK2β HK-2 cells) decreased the levels of Akt pS129 (Fig 1A and ref. [7]). This suggests that CK2 holoenzyme is required for Akt phosphorylation in cultured cells; alternatively, since CK2β-downregulation in HK-2 cells is accompanied by a concomitant decrease in CK2α’ (Fig 1A and ref. [7]), the reduced Akt S129 phosphorylation could be due to a different ability of CK2α and CK2α’ to recognize Akt as substrate. To discriminate between these possibilities, we decided to perform a study by means of a further characterization of CK2 activity in shCK2α/β cells and an *in vitro* analysis with recombinant enzymes.

To better define CK2 activity in shCK2α and shCK2β compared to shCV cells, we analysed the phosphorylation of eukaryotic translation initiation factor eIF2β, whose Ser2 is a well-known specific type III CK2 substrate [38] which is phosphorylated by the holoenzyme but not by the isolated catalytic subunits [35,39]. Fig 1B shows that eIF2β pS2 levels decreased slightly in shCK2α HK-2, according to the partial reduction of CK2β (Fig 1A and reference [7]), while a much stronger decrease was observed in shCK2β HK-2 cells. Next, the general pattern of CK2 endogenous substrates was analysed by means of an antibody that recognizes endogenous proteins containing a pS/pTDXE motif, corresponding to the CK2 phosphorylation consensus sequence. Downregulation of CK2α, accomplished by the constitutive expression of shCK2α, affected only in part the intensity of most of the bands, what suggests that the CK2 forms resulting from combination of CK2α’ with the remaining CK2α and CK2β were sufficient to sustain their phosphorylation (Fig 1C). In contrast, the intensity of certain phosphoprotein bands (i.e. bands 2 and 3) was strongly decreased in shCK2β cells. These data support the existence of substrates highly specific for CK2 holoenzyme in HK-2 cells.

**Fig 2:**
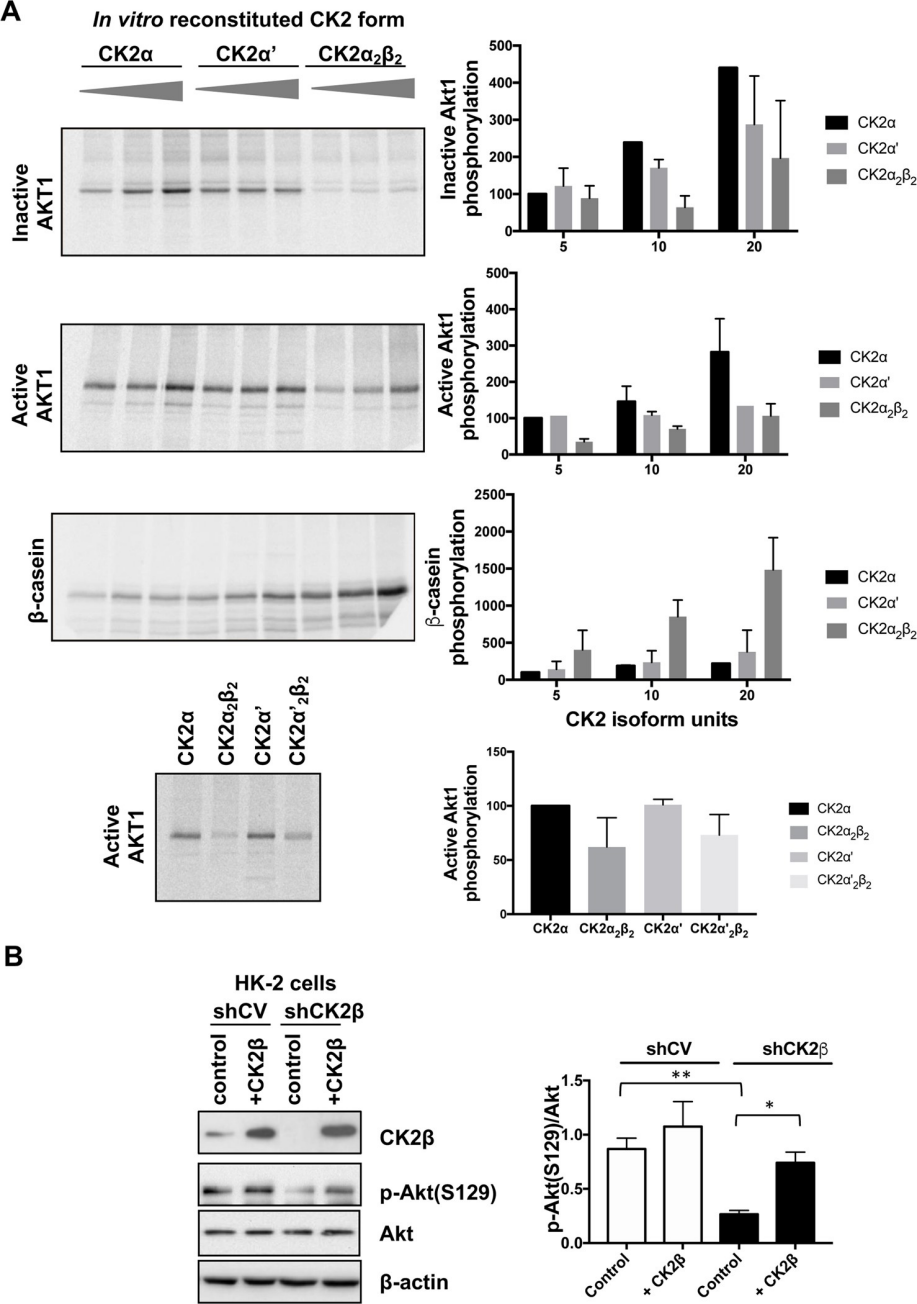
Phosphorylation of Akt by different forms of CK2. (A) Increasing amounts of CK2α (1.25, 2.5 and 5 ng), CK2α’ (40, 80 and 160 ng) or CK2α2β2 (2.5, 5 and 10 ng), corresponding to equal activity towards CK2-tide peptide, were incubated with 0.3 μg of active Akt1 or 0.3 μg of inactive Akt1, or 1 μg β-casein as a control. In the right panel, 0.3 μg of active form of Akt was incubated with CK2α (1.25 ng), CK2α2β2 (5 ng), CK2α’ (40 ng) or CK2α’2β2 (25 ng) in the presence of 150 mM NaCl. The amount of each enzyme used was equally active against the CK2 specific peptide (CK2-tide: RRRADDSDDDDD) as determined by kinase activity assays (not shown). Upon radioactive phosphorylation, proteins were separated by SDS-PAGE. A representative digital autoradiography of the dried gel is shown. The bar graph on the right shows the relative quantitation of the bands (means ± SEM, n = 3), performed by analysis with CyclonePlus Storage Phosphor System, (PerkinElmer); the unit amounts of each isoform are indicated, and activity is reported as % of that measured with 5 units of CK2α. (B) shCV HK-2 and shCK2β HK-2 were transiently transfected with CK2β pCMV-HA vector. Representative Western blot analysis with the indicated antibodies is shown. β-actin was used as a loading control. Quantification (p-Akt(S129)/ AKT) is shown on the bar graph on the right (means ± SEM, n = 2).

Then, the ability of the distinct CK2 forms to phosphorylate Akt1 was assayed in cell-free *in vitro* assays. For this purpose, recombinant CK2α, CK2α’, CK2 holoenzyme α_2_β_2_ or α’_2_β_2_ were used to phosphorylate Akt1. This was provided in its inactive or active forms, meaning that the canonical activation sites T308 and S473 were present in a phosphorylated (active Akt) or unphosphorylated (inactive Akt) status. We used different amounts of each CK2 isoforms, corresponding to the same units calculated by using the CK2-tide peptide as model substrate (see Materials and Methods). To be sure that these amounts corresponded to similar activity also towards protein substrates, the different isoforms were also tested towards β-casein (Fig 2A lower panel). Our results show that both CK2α and CK2α’ phosphorylate either active or inactive Akt1, while the holoenzymes (α_2_β_2_ or α’_2_β_2_) were even less efficient, especially towards inactive Akt1 (Fig 2A) (despite a higher activity towards the model protein substrate β-casein). These results indicate that the decrease in Akt pS129 observed in shCK2β cells is not due to differences in the intrinsic ability of potentially free CK2α and CK2α’ subunits to phosphorylate Akt, but to cellular conditions that make this reaction dependent on CK2β.

In order to confirm the requirement of CK2β for efficient Akt S129 phosphorylation in cells, we tested the effect of restoring CK2β expression in shCK2β HK-2 cells. Fig 2B shows that the forced expression of CK2β in shCK2β cells restored Akt pS129 to a level similar to control shCV cells. These results are in support of the need of CK2 holoenzyme for Akt1 phosphorylation in cultured HK-2 cells.

The dependence of Akt pS129 on CK2β is not cell-specific: we found that pS129 level decreased in all cells were CK2β amount was reduced or knocked out, such as in HEK293T and C2C12 (S1A and S1B Fig) and GN11 (not shown, manuscript in preparation).

### 3.2 CK2β depletion in the renal cell line HK-2 unbalances p-Akt(T308) / p-Akt(S473) ratio

CK2 activity is known to heavily impact on Akt signalling, by means of an intricate network of connections either dependent or independent on the phosphorylation of S129 in Akt1 [15]. The observations described so far of a strong effect of CK2β knock-down on Akt pS129 prompted us to continue our characterization on how the silencing of different CK2 subunits affects signalling pathways in HK-2 cells, with special reference to PI3K/Akt signalling.

**Fig 3:**
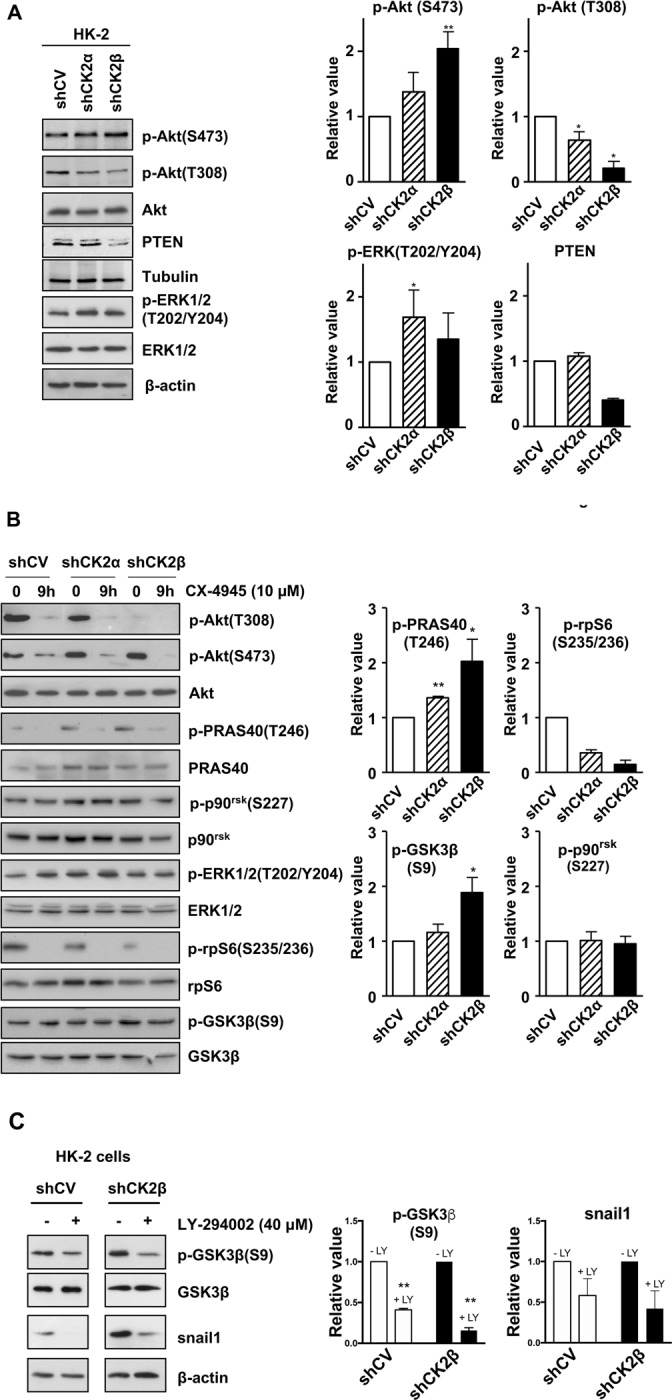
Effect of CK2 subunit down-regulation and CK2 inhibition on ERK and Akt signalling pathway components. (A) HK-2 control and silenced cells were lysed, and protein extracts were analysed by western blot using the indicated specific antibodies. Tubulin and/or β-actin were used as loading controls. (B) HK-2 control and silenced cells were seeded and treated with 10 μM of CX4945 for 9 h, then lysed and protein extracts analysed by western blot with the indicated antibodies against components of the PI3K/Akt/mTOR and ERK1/2 signalling pathways.

First, we analysed the phosphorylation level of T308 and S473, the main Akt activation sites. We found that pT308 was strongly decreased in shCK2β cells, while in shCK2α cells the decrease was more moderate (Fig 3A). In contrast, pS473 slightly and markedly increased after CK2α and CK2β silencing, respectively (Fig 3A). In order to know if these strongly opposite effects on Akt sites observed in shCK2β HK-2 cells were cell-specific, we analysed other CK2β-defective cells. We found that the effects were observable also in other cell types, but they required a stable reduction of CK2β expression. In fact, when we obtained a transient CK2β downregulation by siRNA strategy in HEK293T cells and even in HK-2 cells, we did not observe any significant change of the Akt activation phospho-sites (S1A Fig). On the contrary, by analysing C2C12 cells (S1B Fig) or GN11 (not shown, manuscript in preparation) where CK2β was stably knocked out by CRISPR-Cas9 technology, we found that pS473 was increased, while Akt pT308 was strongly decreased. The reduced pT308 levels were not due to a reduced activity of PDK1, as judged by the level of its activating phosphorylation site pS241 (S2 Fig). As shown in Fig 3A, downregulation of CK2β in HK-2 cells also caused a drop in PTEN, the lipid phosphatase which deactivates Akt pathway through dephosphorylation of PIP3 into PIP2. This finding is explainable considering that CK2 is known to protect PTEN from proteasomal degradation [20], and is also in possible agreement with the observed hyperphosphorylation of pS473. On the other hand, it is also known that inhibition of CK2, while decreasing PTEN stability, increases its lipid phosphatase activity [21]. Therefore, the effects of CK2 targeting on PTEN functions might not be simplistically predicted. However, a clear indication coming from our results is that the regulation of PTEN amount depends on CK2 holoenzyme, since the reduction of CK2α is not sufficient to significantly alter it.

The PI3K/Akt and ERK1/2 pathways are tightly interconnected [22,30], and both are modulated by CK2 [14]. Therefore, we were interesting to knowing if also ERK1/2 signalling was influenced by changes in CK2 subunits; as shown in Fig 3A, we found that ERK1/2 phosphorylation at the activating sites pT202/pY204 was not significantly affected by downregulation of either CK2α or CK2β.

Then, we performed further investigation by treating HK-2 cells with the CK2-inhibitor CX-4945 [40]. Considering that not-tumour cells are quite poorly sensitive to CK2 inhibition [12,40,41], the time and dose conditions were chosen in order to be sure that CK2 was inhibited, but apoptosis symptoms were not induced (not shown). Under these conditions, we found that CX-4945 strongly reduced the phosphorylation of both Akt T308 and S473 sites (Fig 3B).

There was therefore a sharp discrepancy between the effect on pS473 induced by the inhibition of CK2 catalytic activity and the silencing of its subunits. This was not cell type-dependent, since a CX-4945-induced reduction of pS473 Akt, besides pT308, was repeatedly reported by us and other groups (e.g. [40,42–45] and was also observed in C2C12 (S1C Fig) and in GN11 (not shown) cells, where we found that CK2β knock out caused instead an increased pS473 level. The reduction of pS473 by CX-49445 was observed also in shCK2β, suggesting that its increase due to CK2β downregulation requires the presence of active CK2 (see Discussion). The phosphorylation of ERK1/2 pT202/pY204 was not blocked by CX-4945, similarly to what observed in response to CK2 subunits silencing. We also analysed p90rsk pS227, a direct substrate of PDK1 (which phosphorylates p90rsk after ERK1/2 [46,47]), and we found that it was essentially unchanged in all cases (Fig 3B). This suggests that PDK1 activation does not require active CK2.

The results presented so far prompted us to study how Akt-downstream events are affected by the different Akt site phosphorylation pattern. To this purpose, we analysed the phosphorylation status of some Akt targets, namely PRAS40, rpS6, and GSK3β. PRAS40 is a direct substrate of Akt, whose phosphorylation at T246 has been reported to depend on mTORC2-mediated Akt S473 phosphorylation in platelet [27]. Indeed, we found that PRAS40 T246 phosphorylation was increased in shCK2β cells and strongly inhibited by CX-4945, thus mirroring the phosphorylation levels of Akt S473 (Fig 3B).

The ribosomal S6 protein becomes phosphorylated at S235/S236 after activation of both RAS/ERK1/2/p90rsk and PI3K/Akt/mTORC1/S6K signalling cascades [48]. We found that basal rpS6 pS235/pS236 levels were reduced in shCK2α and more markedly in shCK2β cells, and completely abolished by CX-4945 (Fig 3B). This mirrors the level of Akt pT308, but not of p90rsk activation.

On the contrary, when we checked Ser9 in GSK3β, another converging point of Akt and ERK1/2 pathways, being targeted by Akt but also by p90rsk, we found that this site was increased by CK2 subunits silencing (CK2β in particular), but little affected by CX-4945 (Fig 3B). These results suggest that, under our experimental conditions, PI3K/Akt/mTORC1/S6K is the major regulator of rpS6 phosphorylation, while GSK3β Ser9 phosphorylation is mainly controlled by ERK1/2/p90rsk pathway.

The intensity of the bands detected by Western blot corresponding to the indicated phospho-proteins were quantitated by densitometry, normalized to the intensity of the band of their non-phosphorylated form, and the mean values of the changes (± SEM) are shown in the bar graphs on the right. The number n of experiments used for the quantification was: n = 4 for p-Akt (S473), n = 4 for p-Akt (T308), n = 6 for p-ERK (T202/Y204), n = 2 for PTEN, n = 3 for p-PRAS40 (T246), n = 2 for p-rpS6 (S235/236), n = 7 for p-GSK3β (S9), n = 4 for p-p90rsk (S227). When n = 2, statistical significance was not calculated. (C) HK-2 control and CK2β-silenced HK-2 cells were treated with 40 μM LY-294002 for 8 h. Protein extracts were then analysed for Snail1, p-GSK3β Ser9 and total GSK3β by western blot, using specific antibodies. β-actin was used as the protein loading control. Representative results are shown; the bar graphs on the right shows the mean values of the changes (± SEM, n = 2) compared to each untreated control cells; the intensity of p-GSK3β was normalized to GSK3β, snail1 to actin.

### 3.3 Decreased p-GSK3β(S9) level in CK2β-downregulated HK-2 cells affects Snail1 levels

Snail1 is a transcription factor involved in the control expression of epithelial-to-mesenchymal transition (EMT) genes and its protein stability is negatively affected through hierarchical phosphorylation by CK2 and GSK3β, a process reported to strongly depend on CK2β levels [32]. In a previous work we observed that CK2β downregulation increased Snail1 levels in HK-2 [7]. However, the increases in GSK3β(S9) phosphorylation observed now in CK2β downregulated HK-2 cells led us to re-evaluate the need of CK2β in the events that control Snail1 stability. The PI3K/AKT inhibitor LY294002 is known to decrease GSK3β phosphorylation and suppresses EMT in HK-2 [49]. We found that exposure to LY294002 decreased p-GSK3β(S9) levels in both control and CK2β downregulated HK-2 (Fig 3C). Moreover, this treatment also caused a decrease in Snail1 levels, both in control and CK2β downregulated HK-2 cells.

## 4. Discussion

**Fig 4:**
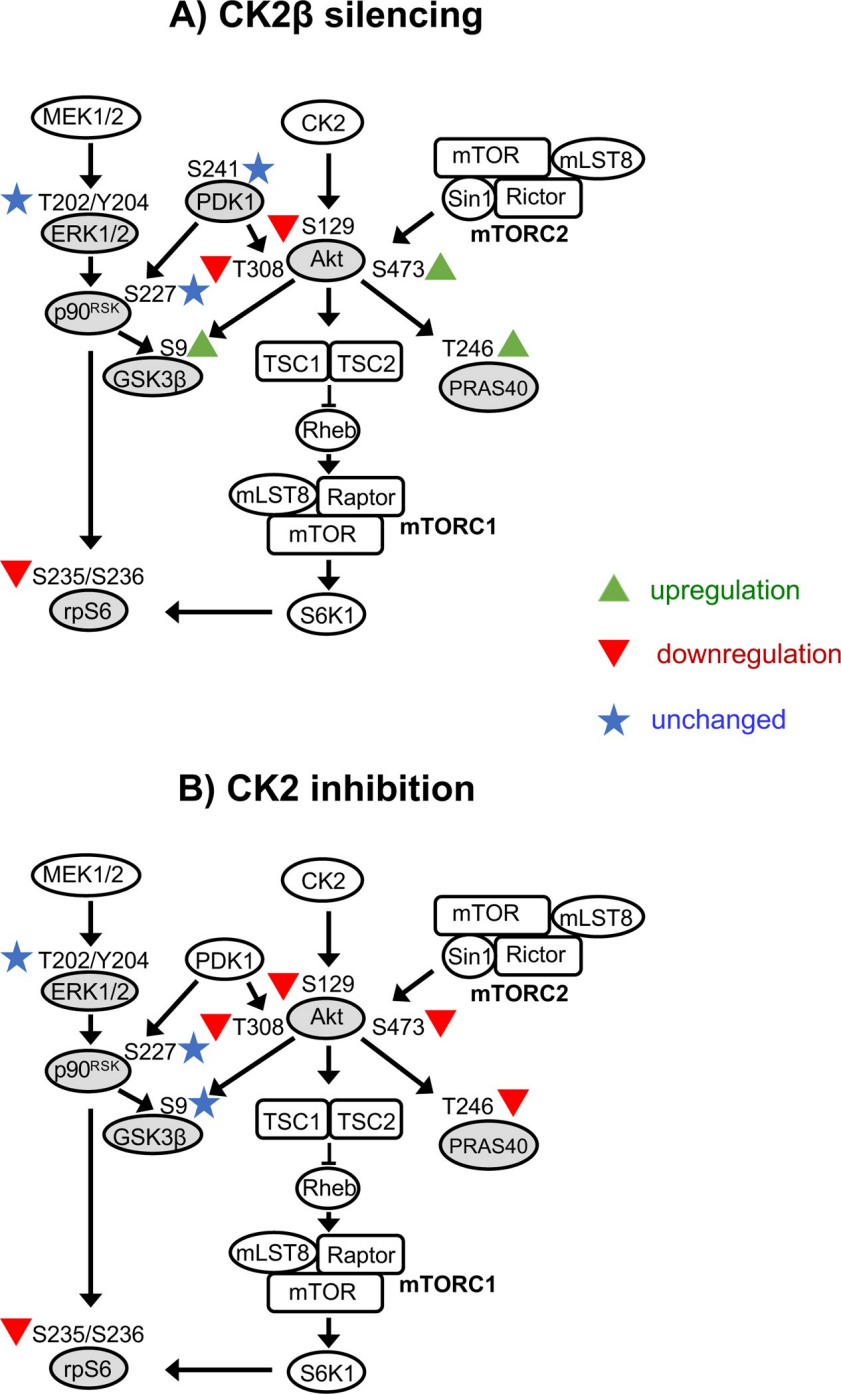
Diagram showing some potential interactions of CK2 with proteins involved in the PI3K/Akt/mTOR and ERK1/2 pathways. The components whose specific phosphorylation has been checked in this work are indicated in grey. The effects of CK2β silencing (A) or CK2 inhibition by CX-4945 (B) in HK-2 cells are highlighted by colours (upregulation in green, downregulation in red, unchanged in blue). The effect of CX-4945 on Akt pS129 is inferred from previous work of our and other laboratories (e.g. [18,40]).

With this study, we investigated how CK2 subunits and activity are involved in the regulation of PI3K/Akt and ERK1/2 signalling pathways in the normal renal tubular epithelial HK-2 cells. A scheme of the analysed signalling proteins is depicted in Fig 4, which also highlights those components altered specifically by CK2β knockdown (Fig 4A) or by global CK2 inhibition (Fig 4B).

Our results support the need of active CK2 to sustain Akt phosphorylation at the activating sites T308 and S473 in HK-2 cells. A considerable part of our study has been performed in cells where the levels of CK2β were down-regulated. It must be kept in mind that CK2 is active in the absence of the regulatory β subunit [50], and indeed the activity on CK2-tide, the specific CK2 substrate peptide, is not altered by CK2β downregulation in HK-2 cells [7]. However, reduced CK2β levels are expected to alter the CK2 substrate selection. In fact, three kinds of CK2 substrates are known [50]: the so-called Type I substrates, which are equally phosphorylated by the holoenzymes and the free catalytic subunits, the Type II substrates, phosphorylated by free CK2α or CK2α’ but not by the holoenzyme, and Type III substrates, better phosphorylated by the holoenzyme; these latter are those expected to be affected by CK2β knocking down. Consistently, here we show that the phosphorylation of eIF2β S2, a model of Type III CK2 substrates [39,51], is markedly reduced in CK2β-downregulated cells (Fig 1C). Interestingly, our data indicate that Akt S129 should be considered a sort of Type III CK2 substrate, since we found that CK2β is required for its efficient phosphorylation in HK-2 cells. This is confirmed also in other cells (S1 Fig) and is in accordance to what recently reported [37,52]. However, the requirement of the β subunit is dependent on the cellular environment, since our *in vitro* assays indicated that free CK2α and CK2α’ are both efficiently able to phosphorylate Akt (Fig 2A). Our *in vitro* experiments also indicate that the decreased Akt pS129 level in response to CK2β reduction is unlikely attributable to the decreased CK2α’ level detected in shCKβ HK-2 cells (Fig 1) and in CK2β-knockout C2C12 cells (S1B Fig), being CK2α also able to phosphorylate Akt S129. Taken together, our results suggest the involvement of cellular factors in determining the ability of CK2 to phosphorylate Akt in living cells; their identification will deserve future studies. Data with recombinant enzymes showed that all CK2 isoforms phosphorylated both active or inactive Akt, as previously found [16]. However, the possibility exists, and is under investigation, that previous Akt phosphorylation present in active Akt affects subsequent S129 phosphorylation, in a manner dependent on a specific CK2 isoform (unpublished observation).

A major finding of our work with shCK2β HK-2 cells is the uncoupling of the two crucial Akt activation sites, being the reduced pS129 phosphorylation accompanied by a strong reduction of pT308 and a concomitant increase of pS473. This was also observed in other cell lines (S1B Fig), but it required a permanent CK2β silencing, while transient reduction by siRNA technique was not effective (S1A Fig). This might mean that the altered Akt phospho-sites are the result of a more global rearrangement of cellular signalling: pT308 and pS473 unbalance might represent an adaptive cell response to survive in a context of CK2β deficiency, requiring conditions not compatible with the short time interval of a transient CK2β reduction by siRNA.

Our data indicate that the form of CK2 present in the cells, by impacting on the Akt phospho-sites balance, has important consequent effects on the phosphorylation of Akt substrates. The finding is particularly relevant considering that unbalanced CK2 subunit expression has been suggested to occur in cancer [53,54], with the possible existence of free catalytic subunits in tumour cells.

Although we do not have an obvious explanation for the opposite effects on Akt pS473 and pT308 observed in shCK2β HK-2 cells, a negative impact specifically on pT308 was somehow expected. In fact, we have previously demonstrated that pS129 hampers Akt association to Hsp90/cdc37 chaperone/cochaperone proteins, a requisite for subsequent pT308 dephosphorylation by PP2A phosphatase [15,17]. Since CK2β silencing strongly reduces pS129, it is conceivable that pT308 is more prone to dephosphorylation in shCK2β cells. Another possibility could be a direct effect of CK2β knockdown on PDK1, the enzymes catalysing T308 phosphorylation. However, our results suggest that PDK1 is not affected, as judged by the phosphorylation of its other substrate p90rsk (Fig 3B) and of its activation site (S2 Fig).

The lipid phosphatase PTEN, which dephosphorylates PIP_3_ to PIP_2_ and deactivates the PI3K/Akt pathway, was decreased (Fig 3A), and this could intuitively explain a higher pS473 phosphorylation found in shCK2β HK-2 cells, but barely justifies a lower pT308 level (even considering that CK2 has two paradoxical opposite effects on PTEN functionality, namely protection of PTEN protein from degradation and inhibition of its catalytic activity [20,21]).

Although CK2 might seem to be not required for Akt S473 phosphorylation, as judged by the effect of CK2 subunit silencing (Figs 3A and 1S), the experiments of cell treatment with CX-4945 highlighted that also Akt S473 phosphorylation requires the presence of active CK2, and even its marked increased in shCK2β cells is dependent on CK2 activity (Fig 3). Akt S473 is targeted by mTORC2, whose activity in cells is affected by multiple mechanisms, such as its feedback inhibition by the mTORC1/S6K1 axis, which involves the phosphorylation of the mTORC2 components Rictor and Sin1 [22,55]. Our present data do not allow to draw any clear-cut conclusion on the mechanism by which CK2 impact on pS473. However, when we tried to dissect the contribution of different pathways, we found that rpS6 phosphorylation decreased in CK2-downregulated HK-2 cells, but this was not accompanied by significant changes in p90rsk pS227. On the other hand, CK2 is known to phosphorylate the mTOR complexes proteins Tel2 and Tti1 what is required for their SCFFbxo9 ubiquitin ligase targeting specifically within mTORC1, and results in the attenuation of mTORC1 signalling while sustaining mTORC2 signalling through relief of feedback inhibition [56]. Therefore, the changes in Akt phosphorylation in response to alterations in CK2 subunits might also result from more direct effects of CK2 on components of mTOR complexes.

Another important finding of our work is the different effects that CK2 inhibition or CK2β down-regulation exert on Akt phosphorylation sites and downstream targets (Fig 4). In fact, the CK2 inhibitor CX-4945, while reducing the phosphorylation of all the Akt sites, also prevents that of the Akt targets rpS6 and PRAS40; on the other hand, the knock down of CK2β, which did not reduce and even increased pS473, similarly affected PRAS40 phosphorylation. This allows to conclude that the two Akt substrates rpS6 and PRAS40 have different dependence on the Akt sites, in our experimental conditions: rpS6 phosphorylation correlated with Akt pT308 levels, while PRAS40 phosphorylation more strictly depended on Akt pS473 levels.

As far as rpS6 is concerned, it is worth to note that a correlation between rpS6 pS235/pS236 and Akt pT308 has been previously reported by a study with knock-in mice expressing a PDK1 mutant which produced a very low Akt T308 phosphorylation level (with regular Akt S473): they found that changes in rpS6 pS235/pS236) levels correlated with those of Akt pT308 and S6K1 activity, but not with p90rsk activation [57].

Concerning PRAS40, it was reported that mTORC2-mediated Akt(S473) phosphorylation is essential for PRAS40 T246 phosphorylation both in human platelets and in rat adipocytes [27]. In contrast, Akt pT308 levels, but not Akt pS473, were found to correlate with PRAS40 T246 phosphorylation in human non-small cell lung cancer tumour samples [25].

Also in the case of GSK3β pS9, different findings have been reported, possibly dependent on the cell type. Akt pS473 was found dispensable for GSK3β S9 phosphorylation in human platelet [25]; however, tunicamycin-induced ER stress in human choriocarcinoma JEG-3 cells led to reduced Akt phosphorylation at T308, but increased it at S473, what resulted in increased GSK3β S9 phosphorylation [58]. In our experiments, GSK3β pS9 seems to be mainly under the control of ERK1/2/p90rsk instead of Akt signalling (as already found in different cells by other groups (e.g. [59,60]); however, the concomitant increases in Akt pS473 and GSK3β pS9 levels detected in CK2β-downregulated HK-2 cells are in support that Akt pS473 can sustain GSK3β S9 phosphorylation when Akt T308 phosphorylation is low.

The consequences of CK2 downregulation in HK-2 cells on GSK3β pS9 levels and its connection with Snail1 levels deserves a further comment. GSK3β S9 phosphorylation and deactivation is known to regulate the expression of Snail1 protein induced by TGF-β1 in HK-2 cells [49]. Moreover, GSK3β phosphorylation and destabilization of Snail1 requires the priming phosphorylation of Snail1 by CK2, a process reported to be dependent on CK2β [32,61]. However, we have now observed that the LY294002 induced decrease in GSK3β S9 phosphorylation was accompanied by a decrease in Snail1 levels both in control and in CK2β downregulated HK-2 cells. Therefore, our results indicate that the effect of CK2β downregulation on Snail1 stability in HK-2 cells might be, at least in part, indirect and exerted through changes in Akt-mediated GSK3β S9 phosphorylation.

In summary, with this work we have disclosed important effects of CK2β down-regulation in HK-2 cells, which are related to an unbalanced phosphorylation level of Akt activation sites. This allowed us to demonstrate that, in these cells, rpS6 phosphorylation is under the control of PI3K/Akt signalling, and in particular of Akt pT308 level, while the Akt substrate PRAS40 depends on Akt pS473 level. On the other side, GSK3β phosphorylation seems to be mainly controlled by the ERK1/2/p90rsk pathway in these cells. Finally, our study highlights how reducing CK2 subunit levels or inhibiting CK2 catalytic activity might have profoundly different consequences in CK2-dependent signalling pathways; this points to the possibility that CK2β might have functions that are independent of its participation to the CK2 holoenzyme and are not related to the catalysis process, as already suggested [9,10,61]. Further studies will be necessary to investigate on this possibility.

This study was aimed at investigating the regulatory role of different CK2 isoforms on a the specific Akt1 isoform in HK-2 cells; while there are strong evidences that Akt2 is not a CK2 substrate [18,19], the possibility still exists that Akt3 is phosphorylated by CK2. However, Akt3 does not contain a site homolog to Akt1 S129, therefore phosphorylation would in case occur at not related site(s). Investigation on the possible Akt3 regulation by CK2 could be of future interest especially for those kinds of cells where this isoform, less ubiquitous than Akt1 and Akt2, is particularly highly expressed.

## Supporting information

S1 Fig Effect of CK2β subunit down-regulation and CK2 inhibition in different cells. (A) CK2β transient down-regulation was performed by siRNA for 72h in HEK293T and HK-2 cells. Control and silenced cells were lysed, and protein extracts were analysed by western blot using the indicated specific antibodies (B) CK2β stable knock out (KO) was performed by CRISPR-Cas9 in C2C12 cells. Control and silenced cells were lysed, and protein extracts were analysed by western blot using the indicated specific antibodies. (C) WT C2C12 cells were treated with 10 μM of CX4945 for 9 h, then lysed and protein extracts analysed by western blot with the indicated antibodies.
(TIF)

S2 Fig Effect of CK2 targeting on PDK1 activation. Lysates from cell extracts obtained as in Figs 3A and S1 were analysed by western blot using anti-p-PDK1(S241) antibodies. Tubulin or β-actin were used as loading controls.
(TIF)

## Abbreviations

Akt: Ser/Thr protein kinase Akt; also called PKB
CK2: protein kinase CK2, formerly known as casein kinase 2
EMT: Epithelial-to-mesenchymal transition
GSK3β: Glycogen Synthase Kinase 3β
mTORC1: mTOR complex 1
mTORC2: mTOR complex 2
PDK1: 3-phosphoinositide-dependent protein kinase-1
PI3K: Phosphatidylinositl 3-kinase
PRAS40: Proline-Rich Akt Substrate of 40 kDa
PTEN: Phosphatase and TENsin homologue deleted in chromosome TEN
p90rsk: p90 ribosomal S6 protein kinase, also known as RSK
S6K1: p70 ribosomal S6 protein kinase
TSC2: Tuberous sclerosis complex 2
