# Supplementary figures and images for: Effects of CK2β subunit down-regulation on Akt signalling in HK-2 renal cells
Source: PLoS One. 2020 Jan 7;15(1):e0227340. doi: 10.1371/journal.pone.0227340 (PMC6946142; doi:10.1371/journal.pone.0227340)

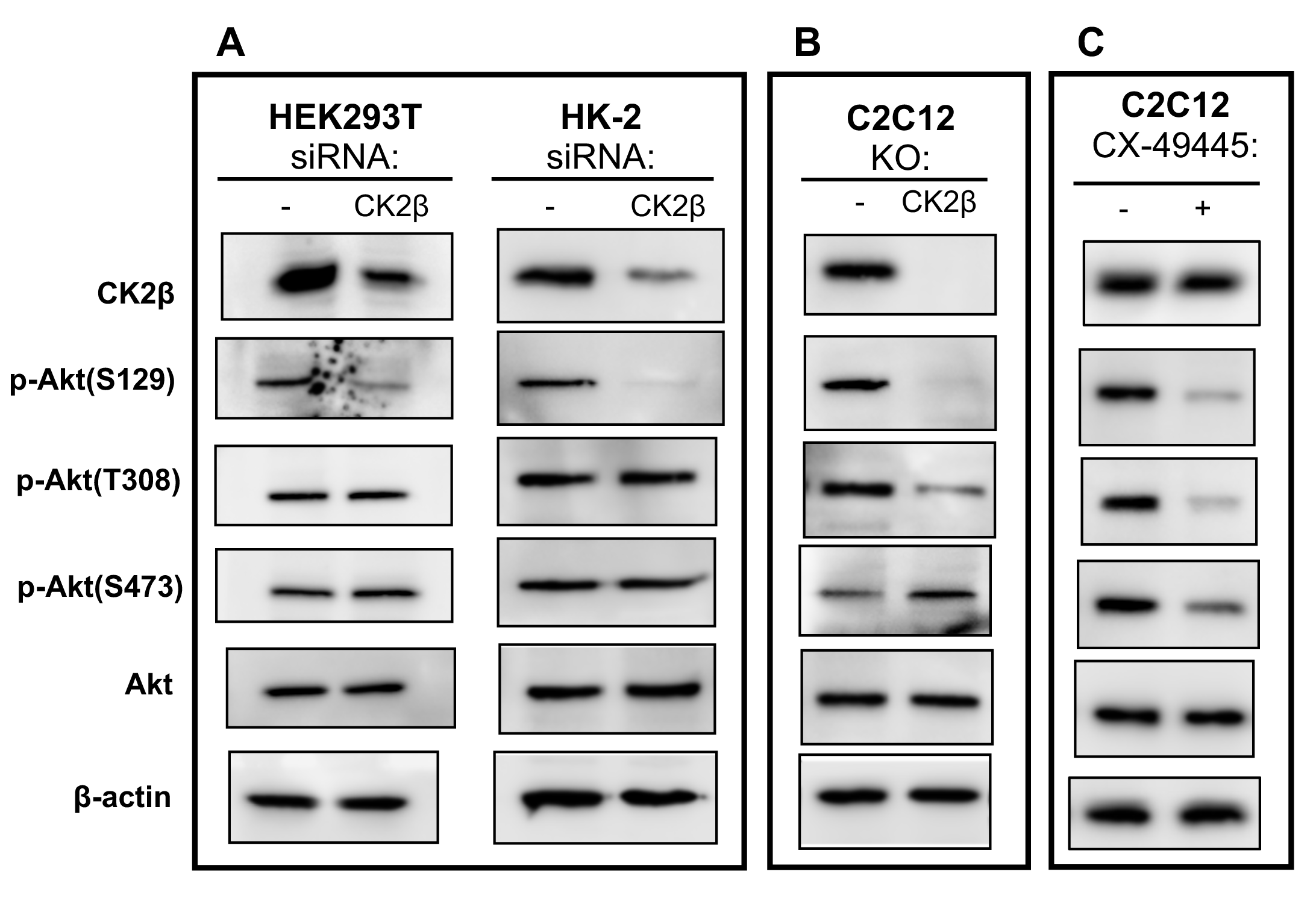

Supplement: S1 Fig — (A) CK2β transient down-regulation was performed by siRNA for 72h in HEK293T and HK-2 cells. Control and silenced cells were lysed, and protein extracts were analysed by western blot using the indicated specific antibodies (B) CK2β stable knock out (KO) was performed by CRISPR-Cas9 in C2C12 cells. Control and silenced cells were lysed, and protein extracts were analysed by western blot using the indicated specific antibodies. (C) WT C2C12 cells were treated with 10 μM of CX4945 for 9 h, then lysed and protein extracts analysed by western blot with the indicated antibodies. (TIF) [file pone.0227340.s001.tif]

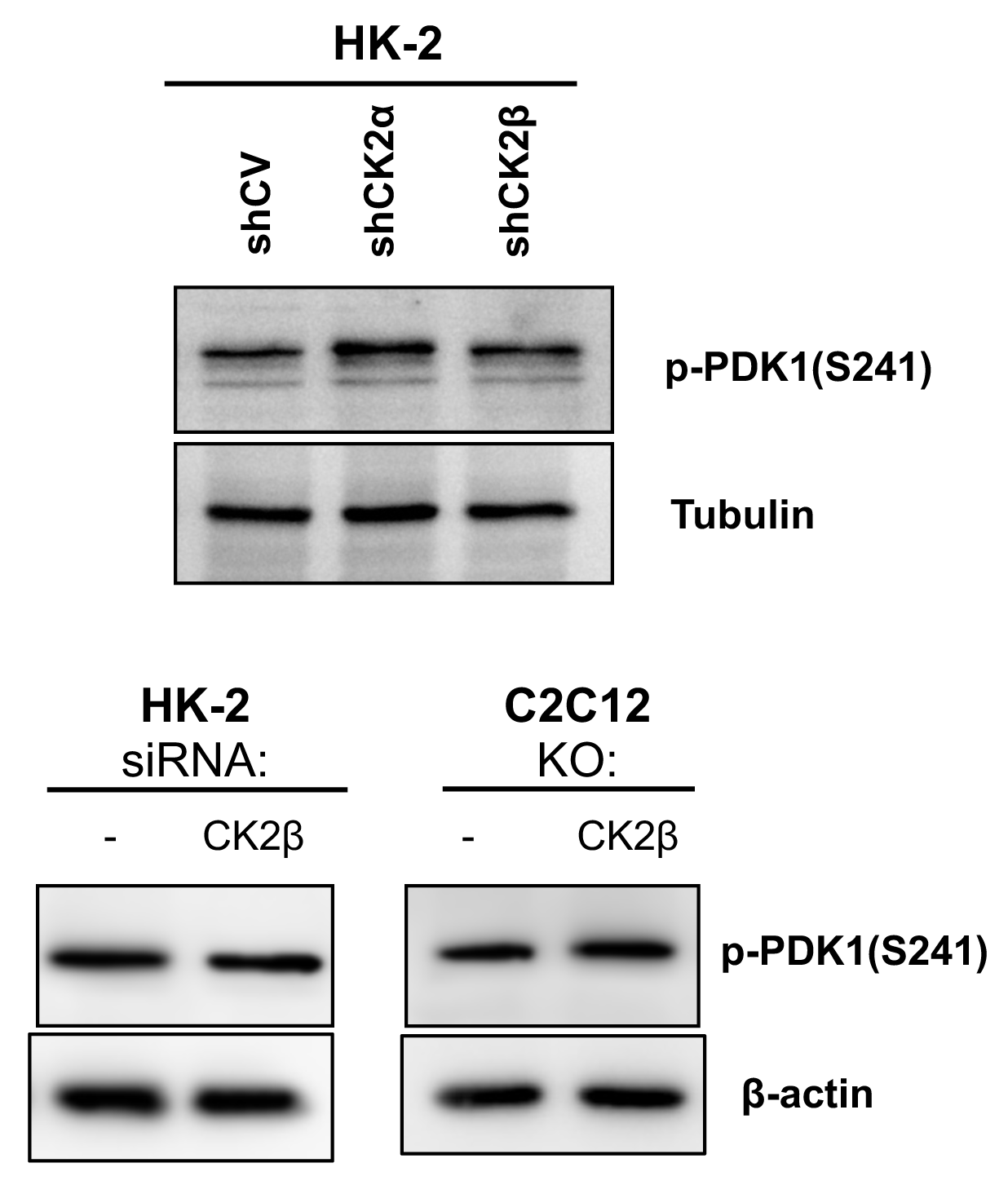

Supplement: S2 Fig — Lysates from cell extracts obtained as in Figs 3A and S1 were analysed by western blot using anti-p-PDK1(S241) antibodies. Tubulin or β-actin were used as loading controls. (TIF) [file pone.0227340.s002.tif]
